# Impact of UGT1A9 Polymorphism on Mycophenolic Acid Pharmacokinetic Parameters in Stable Renal Transplant Patients 

**Published:** 2013

**Authors:** Talia Mazidi, Mohammad-Reza Rouini, Mohammad-Hossein Ghahremani, Simin Dashti-Khavidaki, Mahboob Lessan-Pezeshki, Farrokh lagha Ahmadi, Jamshid Salam-Zadeh, Ali Mandegary, Kheirollah Gholami

**Affiliations:** a *Department of Clinical Pharmacy, Faculty of Pharmacy, Tehran University of Medical Sciences, Tehran, Iran.*; b *Professor of Pharmaceutics, Department of Pharmaceutics, Faculty of Pharmacy, Tehran University of Medical Sciences, Tehran, IR Iran.*; c *Associate Professor, Department of Toxicology and Pharmacology, Faculty of Pharmacy, Tehran University of Medical Sciences, Tehran, Iran.*; d *Professor of Medicine Department of Nephrology, Imam Hospital, Tehran University of Medical Sciences, Tehran, Iran.*; e *Assistant Professor of Medicine, Department of Nephrology, Imam Hospital, Tehran University of Medical Sciences, Tehran, Iran.*; f *Associate Professor, Department of Clinical Pharmacy, Faculty of Pharmacy, Shaheed Beheshti University of Medical Sciences, Tehran, Iran.*; g *Assistant Professor, Department of Toxicology and Pharmacology, Faculty of Pharmacy, Kerman University of Medical Sciences, Kerman, Iran. *

**Keywords:** UGT, Polymorphism, Mycophenolate mofetil, Renal transplantation

## Abstract

There are wide individual differences in pharmacokinetic parameters of mycophenolate mofetil (MMF) among transplanted patients. Some studies have shown that single nucleotide polymorphisms (SNPs) of the Uridine Diphosphate Glucuronosyl Transferase1A9 (UGT1A9) are responsible for these differences in early days after transplantation. Therefore it was decided to evaluate the influence of UGT polymorphism on MMF pharmacokinetics among stable Iranian transplant patients.

This was a cross sectional study from March 2008 through December 2008 in Imam Khomeini Hospital affiliated to the Tehran University of Medical Sciences in Iran. Blood samples were taken from 40 *de novo *stable Iranian renal transplant patients taking 2 g MMF daily with Sr_Cr_≤1.4 mg/dL with at least 3 months history of transplantation. Appropriate PCR and HPLC methods were used for the determination of SNPs and their impact on MPA pharmacokinetics.

T-275A polymorphism occurred in 15% of patients, UGT1A9*3 occurred in 2.5% of patients. Carriers of T-275A polymorphism had significant lower MPA AUC _0-12_ in comparison with non-carriers or wild type (73.3±17.8 g/h/mL vs. 110.8±31.1 μg/h/mL, p = 0.006). There was no significant difference in AUC _6-12_ between the two groups although carriers of T-275A SNP had lower MPA AUC _6-12_ (22.4±4.5 μg/h/mL vs. 26.8±10.2 μg/h/mL, p = 0.24). C_max_ was lower in the carriers of (20.2±9.0 μg/mL vs. 37.2±12.5 μg/mL, p=0.004). There was no significant difference in C_0 _between two groups. (3.0±1.2 μg/mL vs. 3.9±1.6 μg/mL, p = 0.1).

This study in Iranian stable transplanted patients shows that carriers of T-275A polymorphism had significantly lower MPA exposure compared to non-carriers.

## Introduction

Based on the National Kidney Foundation’s (NKF) guidelines, chronic kidney disease (CKD) is defined as kidney damage and/or reduced kidney function. Therefore CKD is defined as the presence of evidence of kidney damage with an abnormal glomerular filtration rate (GFR) or alternatively, for at least 3 months, by a GFR < 60 ml/min/1.73 m^2^ (1). End–stage renal disease (ESRD) occurs when GFR decreases to less than 15 ml/min/1.73 m^2^ and dialysis or transplantation are required to remove uremic toxins and maintain homodynamic stability (2). In comparison to dialysis, transplantation has become the treatment of choice for patients with ESRD, with significant improvements in quality of life and physiologic parameters (1). By development of newer immunosuppressive agents, such as cyclosporine, tacrolimus, mycophenolate mofetil (MMF), polyclonal and specific monoclonal antibodies, the incidence and intensity of acute rejection has been reduced (1). According to several clinical trials, MMF therapy has been associated with a 50% reduction in the incidence of acute rejection in the first year after transplantation and to a lesser extent in progression of chronic rejection (1). MMF is classified as an antiproliferative antimetabolite (1, 3, 4). It is the mopholinoethyl ester prodrug of mycophenolic acid (MPA) that selectively, reversibly and noncompetitively blocks inosine monophosphate dehydrogenase (IMPDH) required for proliferation of T and B lymphocytes. MMF is almost completely hepatically metabolized to its glucuronide derivative, mycophenolic acid glucuronide (MPAG), which undergoes enterohepatic circulation and is ultimately excreted renally (1). While a strong relationship between AUC (Area under plasma concentration-time curve) of MPA and prevention of acute rejection has been reported, such co-relation has not been established between C_0 _(Predose plasma concentration) of MPA and clinical outcomes (5, 6). Due to high individual variations in pharmacokinetic parameters of MMF and its interactions with other concomitantly used drugs in renal transplant patients serum concentration monitoring is recommended (5). While co-administration of MMF and cyclosporine decreases the AUC of MPA due to inhibition of enterohepatic circulation (7), MMF interaction with tacrolimus results in inhibition of MPA glucuronidation which consequently increases MPA plasma concentration (8). Interaction between MMF and corticosteroids decreases AUC of MPA due to uridine dipphosphate glucuronosyl transferase (UGT) induction (9). In renal transplant patients, interindividual differences of AUC and C_0 _of MPA have been reported. These differences have been related to gender, time after transplantation, serum albumin concentration, renal function, concomitant drugs and pharmacogenetic factors (6, 10-13). In addition to differences in absorption, distribution and elimination, MMF metabolism is considered to be important in its interindividual differences (14). Several UGT isoenzymes such as UGT1A1, 1A7, 1A8, 1A9 and 1A10 can metabolize MPA to its glucuronide derivative MPAG, among them UGT1A9 has a more important role in hepatic drug metabolism (15). There are great interindividual differences in gene expression and enzymatic activity of UGT in adults (14, 16). Girard and colleagues showed that UGT1A9 protein level varied by 17 fold and glucuronidation activity varied by 9.5 fold in human liver microsomes (17). One of isoforms studied in this regard is UGT1A8 showing that UGT 1A8*3 A^173^ Y^277 ^mutation significantly decreases the enzyme activity (18). The most extensive reports in this area are related to single neucleotide polymorphism (SNPs) discovered in UGT1A1, UGT1A7, UGT1A9 and UGT2B7. The polymorphisms found in UGT1A9 are I399, M^33^T, -2152, -665, -331/-440, -275 (14, 17, 19). Among these SNPs the more important ones are C-2152T and T-275A in promoter region of UGT1A9 that have the most consistent relationship with UGT1A9 expression and level (14). The level of UGT1A9 in carriers of T– 275A SNP was 1.4 times and in carriers of T-275A/ C-2152T SNP was 1.6 times more than non-carriers (14, 17). Although UGT1A9 M^33^T decreases glucuronidation activity of UGT1A9 and increases MPA plasma levels, C-2152T and T-275A polymorphisms decrease the AUC and C_0 _of MPA as a result of increase in glucuronidation activity of UGT and inhibition of entrohepatic recirculation of drug which in turn decreases deglucuronidation of MPAG (14, 17). Among the discovered polymorphism, UGT1A9 I399 has the prevalence of 15% in Japanese population. However studies showed that this polymorphism cannot explain the interindividual differences in MPA pharmacokinetics (19). Therefore this study was designed to investigate the existence of UGT1A9 M^33^T C-2152T and T-275A polymorphism in Iranian renal transplant patients and their probable influences on pharmacokinetic parameters of drug.

## Experimental

*Study protocol*

This was a cross sectional study carried out from March 2008 through December 2008 in Imam Khomeini Hospital affiliated to the Tehran University of Medical Sciences in Iran. Based on previous studies and prevalence of polymorphism reported and statistical calculations, 40 *de novo *Iranian kidney transplant patients were selected for this study (17, 20). Patients, who voluntarily filled a written consent form, received MMF 2 g daily (Cellcept 500 mg tablet, Roche, Switzerland), cyclosporine and prednisolone as their immunosuppressive regimen. A 3 mL blood at 0, 20, 40, 60, 120, 180, 240, 360, 480, 600 and 720 min after taking 1 g of morning dose of MMF was taken from patients. Plasma was separated by centrifugation at 6000 rpm for 10 min. Both plasma and blood cells were kept at -70 °C for pharmacokinetic and pharmacogenetic studies respectively.

*Inclusion and exclusion criteria*

Patients with at least 3-month history of post renal transplantation, approximately normal renal function (Sr_Cr_≤1.4 mg/dL), cyclosporine level between 100-200 ng/mL taking MMF dosage of 1 g every 12 h who were willing to be hospitalized for 12 h and give 11 blood samples were considered eligible. Exclusion criteria were as follows: acute renal failure, current cytomegalovirus (CMV) infection and gancyclovir administration, receiving polyclonal antibodies, elevated liver enzymes (more than 3 times normal upper limit), gastrointestinal adverse effects of MMF such as severe diarrhea needing MMF dose adjustment and diabetes mellitus. 

*Pharmacogenetic studies*

Genomic DNA was extracted from EDTA-treated whole blood by adding distilled water for red blood cell (RBC) lysing; using white blood cell lysing buffer (WBCLB), Tris 10 mM, EDTA 10 mM and NaCl 50 mM ; SDS 10%; proteinase K (Fermentas) and saturated NaCl). After extraction, the DNA concentration was determined by spectrophotometeric method. For identification of UGT1A9*3, UGT1A9 C-2152T, and UGT1A9 T-275A polymorphism, polymerase chain reaction (PCR)-restriction fragment length polymorphism (RFLP) method was used. Forward and reverse primers for C-2152T were 5-TTG AGA CAG AGT CGT GCT GTT T-3 and 5-AGG TCA AGG TGG GCG TAT C-3, respectively. For T-275A, we used 5-TCA GTG CTA AGG GCC TTG TT-3 and 5-CCT GTG CTG CAA TGT TAA GTC TA-3 as forward and reverse primers, respectively. For UGT1A9*3 forward and reverse primers were 5-GTT CTC TGA TGG CTT GCA CA-3 and 5-ATG CCC CCT GAG AAT GAG TT-3, respectively (20). The PCR reaction was performed using 25 L of taq DNA polymerase, RED master mix (Ampliqon-Denmark), 100 ng DNA, 0.5 μM of forward and reverse primers and de-ionized water up to 50 μL.

The following PCR amplification conditions were used for UGT1A9 polymorphisms: 

*-T-275A: *94°C, 4 min, followed by 30 cycles of 94 °C for 30 sec, 55oC for 30 sec and 72 °C for 45 sec and final 72 °C for 10 min;

*-C-2152T: *94 °C for 2 min, then 10 cycles of 94 °C for 30sec,65 °C (-0.5 °C /cycle) for 30 sec and 72 °C for 30sec followed by 35 cycles of 94 °C for 30 sec, 60 °C for 30 sec and 72 °C for 30 sec and finally 72 °C for 10 min. 

-UGT1A9*3: 94 °C for 130 sec, then 10 cycles of 94 °C for 30 sec, 60 °C (-0.5 °C /cycle) for 30 sec and 72 °C for 30 sec, followed by 35 cycles of 94 °C for 30 sec, 55 °C for 30 sec and 72 °C for 30 sec and final 72 °C for 10 min. 

The PCR products for T-275A, C-2152T and UGT1A9*3 were digested with XbaI (Fermentas), MseI (Fermentas) and StyI (Fermentas), respectively. Digestion conditions were as follows: 10 μL of PCR mixture, 3 μL of buffer (10X), 1 μL of enzyme and 16 μL of de-ionized water to reach the final volume of 30 μL. The reaction was incubated for 4 h in 37 °C for XbaI and StyI enzymes and in 65 °C for MseI enzyme. DNA fragments were separated on 3% agarose gels and visualized by ethidium bromide.

*Pharmacokinetic studies*

*-HPLC system*

HPLC system was chosen based on previous study (21); A Knauer (Knauer Company, Germany) 1001 HPLC pump and a Knauer (Knauer Company, Germany) 2600 UV detector were used. Chromatographic separation was performed on a Hamilton PRP-1 reversed phase column (250 mm × 4.6 mm i.d., particle size 10 μm- Hamilton company, Reno, Nevada) with the suitable Hamilton guard column (25 × 2.3 mm i.d., particle size 12-20 μm- Hamilton Company, Reno, Nevada). The mobile phase ingredients, flow rate and retention times were the same as the previous study done by Ahadi and *et al*.( 21). 

Stock solutions of 1 mg/mL MPA and naproxen and all sample treatments were performed as described previously (21). Eleven standard concentrations ranged from 0.1-60 μg/mL were prepared in plasma and linear least square method was applied for calibration curve).

*-Accuracy, precision, limit of quantification (LOQ) and recovery*

Accuracy, Intra-day precision, Inter-day precision, limit of quantification and absolute recovery of MPA were assessed for four QC concentrations (0.25, 1, 12, 40 μg/mL). 

*Statistical analysis: *Statistical analyses were performed using the Statistical Package for Social Scientists (SPSS version 15.0) computer software. A 3-step statistical analysis was designed. At the first step, the preliminary analyses of the Mann-Whitney, student t-test, Spearman’s rank correlation, Kendall’s rank correlation and Pearson correlation were used to assess any relationship between dependent variables, *i.e. *pharmacokinetics parameters, and possible predictive variables including age, sex, bodyweight, UGT1A9 polymorphism, MMF dose, cyclosporine dose, steroid dose, cotrimoxazole dose, CMV infection, serum levels of creatinine, creatinine clearance and time after transplantation. Predictors which had a relationship with the pharmacokinetic variables with a p-value of less than 0.1 in the above mentioned univariate analysis were eligible to be included in the final model building. Thus, at this step, those predictive variables which were highly unlikely to be associated with the dependent variables were determined and excluded from the multivariate analysis. In the second step, any interaction between eligible variables obtained in the first step, was evaluated using appropriate statistical tests. In the case of significant interaction (p < 0.05), a ridge regression analysis was applied instead of ordinary linear multivariate regression analysis to obtain the final model (the 3^rd ^step).

## Results and Discussion

-Demographics and transplantation-related results: All demographics, transplantation-related characteristics and biochemical parameters of patients are summarized in Tables 1 and 2.

**Table 1:** Demographic characteristics of patients (Data are presented as mean ± SD).

**Parameter**		**Value**	**Parameter**	**Value**
*Baseline demographics*			*Biochemical parameters *	
Number of patients		40	Serum Creatinine (mg/dL)	1.2±0.2
Recipient gender (female/male)		11/29	Hemoglobin (g/dL)	14.3±1.2
Body weight (kg)		70.7±11.3	ALT ^b^ (U/L)	27.5±8.5
Age(yr)		39.5±12.3	AST ^c ^(U/L)	22.8±5.8
			Total Bilirubin (mg/dL)	1.2±0.6
Transplantation-related characteristics			FBS ^d^ (mg/dL)	91.3±7.3
Time after transplantation (mo)		41.5±35.6	TG ^e^ (mg/dL)	145.4±57.5
Re-transplantation (no)		4	Total cholesterol (mg/dL)	169.2±36.5
CMV ^a^ infection (no)		10	MMF daily dose ^f^ (g)	2
Polyclonal antibody treatment (no)		5	Steroid dose (mg/day)	5.2±1.3
Type of donor (living/cadaveric)		36 /4	Cyclosporine dose (mg/kg)	1.8±0.4

a: cytomegalovirus, b: alanine aminotransferase, c: aspartate aminotransferase, d: fasting blood sugar, e: triglycerides, f: mycophenolate mofetil

**Table 2:** Comparison of demographic characteristics between carriers of UGT1A9 T-275A SNP and Wild type (Data are presented as mean ± SD).

**Parameters**	**Wild type**	**Carriers**	**p-value**
*Baseline demographics *			
Number of patients	34	6	
Recipient gender (female/male)	8/26	3/3	
Body weight (kg)	70.9 ± 10.9	69 ± 14.7	0.9
Age (yr)	38.4±11.7	46.3±14.6	0.2
*Transplantation-related characteristics *			
Time after transplantation (d)	1155.3±1074.4	1762.5±958.8	
Re-transplantation (no)	4	0	
CMV^a^ infection (no)	40	0	0.1
Polyclonal antibody treatment (no)	5	0	
Type of donor (living/cadaveric)	31/3	5/1	
*Biochemical parameters *			
Serum creatinine (mg/dL)	1.2±0.2	1.1±0.3	0.7
GFR^b^ (ml/min/1.73m2)	83±17.9	72.3+13.4	0.6
Steroid dose (mg/day)	5.2±1.3	5±1.6	0.3
Cyclosporine dose (mg/kg)	1.8±0.4	1.7±0.2	0.8

a: cytomegalovirus, b: Glomerular filtration rate

-Validity of the HPLC method: Eleven point calibration curves for MPA on separate days were linear over the concentration range of 0.1-60 μg/mL. The mean regression line showed the correlation coefficient (r^2^) of 0.9992 with the equation of y = 0.1403x + 0.0002. Absolute analytical recoveries of the assay in four concentrations were between 86**±**1.4 and 104**±**1.5% with a mean of 94.8%. The method proved to be accurate and precise; the accuracy at four concentration levels ranged from 91.6-109%. The within-day and between–days precision ranged from 2.1-7.8% and 1.2-19.3%, respectively.

**Figure 1:**
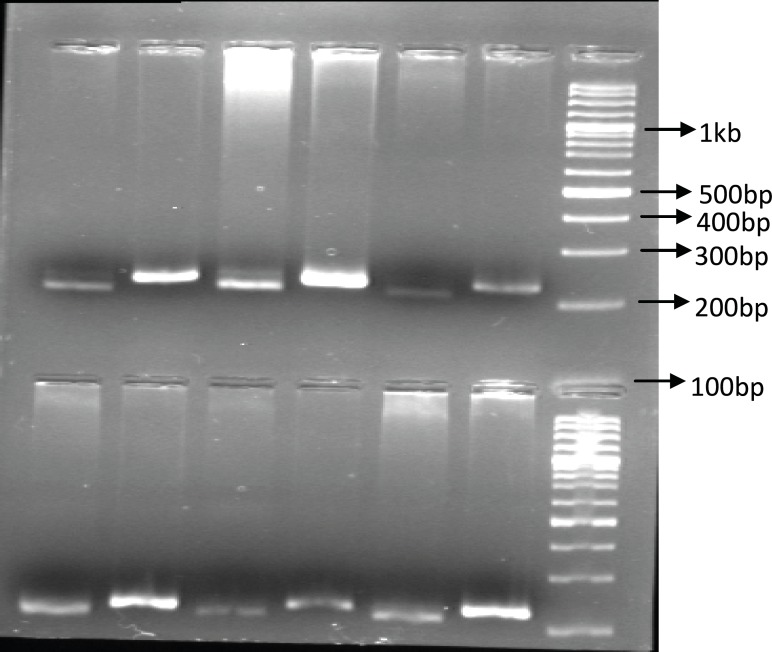
The PCR-RFPL analysis of UGT1A9 T-275A SNP in patients. The DNA from patients was subjected to PCR followed by RFPL using XbaI digest. The reactions were resolved on 2% agarose gel electrophoresis. Lanes 1-3 and 4-6 are from two different patient hetrozygote for T-275A SNP. Lane 1, 4 are PCR products and lane 2, 3 and 5, 6 are XbaI digest of corresponding samples. The reactions were compared to 100 bp DNA ladder. (PCR-RFLP :poly chain reaction-restricted fragment length polymorphism, UGT: Uridine diphosphate glucuronosyl transferase, SNP: Single nucleotide polymorphism).

**Figure 2:**
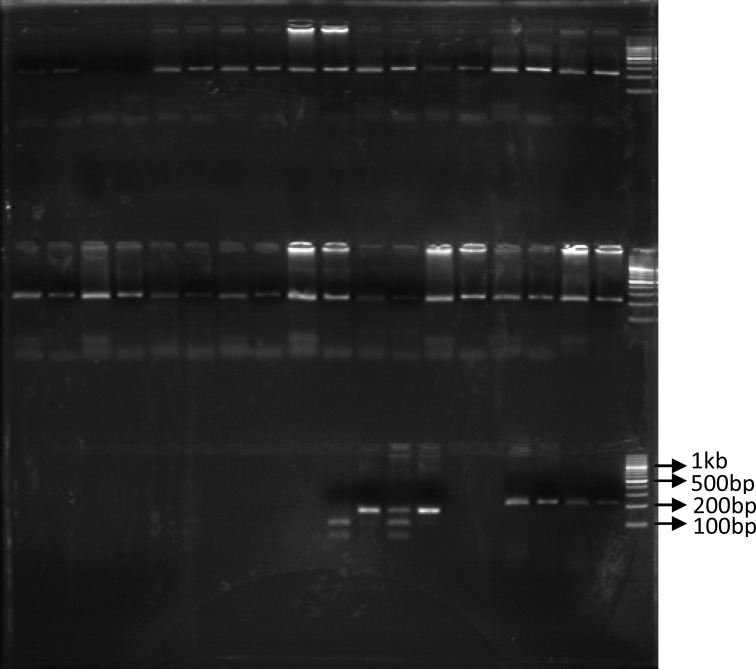
The PCR-RFPL analysis of UGT 1A9*3 T-275A SNP in patients. The DNA from patients was subjected to PCR followed by RFPL using StyI digest. The reactions were resolved on 3% agarose gel electrophoresis. Lanes 1-2 and 3-4 are from two different patients. Lane 1, 3 are PCR products and lane 2 and 3 are StyI digest of corresponding samples. Lane 2 indicates presence of UGT1A9*3 SNP (mut) and Lane 4 indicate wild type UGT1A9 (WT). The reactions were compared to 100bp DNA ladder. (PCR-RFLP: poly chain reaction-restricted fragment length polymorphism, UGT: Uridine diphosphate glucuronosyl transferase, SNP: Single nucleotide polymorphism).

Identification of UGT1A9 polymorphism: In our study population, only 6 patients (15%) were recognized with UGT1A9 T-275A promoter region single nucleotide polymorphism and all of them were heterozygous for this SNP. Among our forty patients only one heterozygous individual carrying UGT1A9*3 mutation was identified (2.5%). Figures 1 and 2 show these identified polymorphisms. 

Impact of UGT1A9 T-275A SNP on pharmacokinetic parameters of MPA: Patients with T-275A polymorphism (6 out of 40) did not differ from wild type in terms of age (p=0.19), bodyweight (p=0.95), steroid dose (p=0.32), cyclosporine dose (p=0.78) and CMV infection (p=0.125).

**Table 3:** Comparison of MPA^a^ pharmacokinetic parameters between carriers of UGT1A9 (Uridine diphosphate glucuronosyl transferase) T-275A SNP^b^ and noncarriers

**Parameters**	**All**	**Noncarriers**	**Carriers**	**p** ***-*** **value**
Number of patients	40	34	6	
AUC_0-12_^c ^(mg.h/L)	104.8±32.3	110.4±31.1	73.3±17.8*	0.007
AUC_6-12_^d^ (mg.h/L)	26.2±9.7	26.9±10.2	22.4±4.5	0.26
C_0_^e^ (mg/L)	3.8±1.6	3.9±1.6	3.0±1.2	0.11
C_max_^f^ (mg/L)	34.6±13.4	37.2±12.5	20.3±9.0*	0.0002
T_max_^g^ (h)	1.0±0.5	0.9±0.5	1.3±0.6	0.22
CL/F ^h ^(L/h)	19. 7±6.2	18.4±5.2	26.9±10.2*	0.002

a: Mycophenolic acid, b: Single nucleotide polymorphism, c: Area under concentration-time curve from 0 to 12 h, d: Area under concentration-time curve from 6 to 12 h, e: Predose plasma concentration, f: Maximum plasma concentration, g: Time to reach maximum plasma concentration, h: Total body clearance.* p ≤ 0.05.

**Table 4:** Determinants of MPA^a^ pharmacokinetic parameters (based on ridge regression results).

**Dependent variables**	**Independent variables**	**r** ^2^	**p-value**
MPA AUC_0-12_^b^ (mg.h/L)	UGT c1A9 T-275A SNP ^d^	0.60(pos)	0.007
MPA AUC_12-6_^e^(mg.h/L)	no covariate retained in the model		
C_0_^f^ (mg /L)	no covariate retained in the model		
C_max_^g^ (mg /L)	UGT1A9 T-275A SNP	0.68(pos)	0.0002
t_max_^h^ (h)	no covariate retained in the model		
CL/F ^i^ (L/h)	UGT1A9 T-275A SNP	0.64(neg)	0.002

a: Mycophenolic acid, b: Area under concentration-time curve from 0 to 12 h, c: Uridine diphosphate glucuronosyl transferase, d: Single nucleotide polymorphism, e: Area under concentration-time curve from 6 to 12 h, f: Predose plasma concentration, g: Maximum plasma concentration, h: Time to reach maximum plasma concentration, i: Total body clearance.

According to our results, patients carrying T-275A SNP had significantly lower AUC_0-12 _for MPA in comparison to the wild type (73.3 ±17.8 mg/h/L versus 110.4±31.1 mg/h/L respectively, p=0.007). The C_max_ (maximum plasma concentration) was significantly lower in the presence of T-275A polymorphism (20.3±9.0 mg/L versus 37.2±12.5 mg/L respectively, p=0.0002), though there was no significant difference in C_0 _between groups (3.0±1.2 mg/L versus 3.9±1.6 mg/L respectively, p =0.11). 

Patients with UGT1A9 T-275A SNP had longer T_max_ (time to reach maximum plasma concentration) in comparison with patients with wild type UGT1A9, although this difference was not statistically significant (1.3±0.6 h versus 0.9±0.5 h respectively). Moreover, even though AUC_6-12 _of MPA in carriers of T-275A SNP, was lower than AUC_6-12 _in wild type group, it was not statistically significant (22.4±4.5 mg/h/L versus 26.8±10.2 mg/h/L respectively, p=0.26). When we compared CL/F (total body clearance) between two groups there was a statistically significant difference between groups in which carriers of UGT1A9 polymorphism had increased clearance compared with wild type patients (26.9±10.2l/h versus 18.4±5.2 L/h respectively, p=0.002). 

Impact of UGT1A9*3 mutations on pharmacokinetic parameters of MPA: We had only one heterozygous carrier of UGT1A9*3, therefore statistical analysis of the data was not possible. Pharmacokinetic parameters of this patient were 85.7 mg/h/L; 28.9 mg/h/L; 2.8 mg/L; 21.2 mg/L; 0.7 h; 21.9 L/h for AUC_0-12_, AUC_6-12_, C_0_, C_max_, T_max_ and CL/F of MPA respectively. Data are summarized in Table3.

Table 4 shows determinant of MPA pharmacokinetic parameters in the final model. Since there is great interindividual variability in AUC_0-12 _of MPA among renal transplant recipients (6,10), many experiments have been designed to examine the effect of different factors such as gender, body weight, pathophysiological factors, comedications, race and pharmacogenetics on MPA pharmacokinetics (11-13). The aim of this study was to evaluate the effect of some reported UGT1A9 SNPs on pharmacokinetic parameters of MPA. Although there are many polymorphisms in other UGTs (18, 19) and UGT1A9 enzyme identified by Girard and Kazuyuki (17, 19) none of them has been reported to increase MPA glucuronidation activity to the same extent as the T-275A and C-2152T mutations. In addition, only Kuypres (20) has reported the frequency of T-275 A and C-2152T SNPs in his study, therefore we could assess only T-275A, C-2152T and UGT1A9*3 in our population.

In accordance with Kuypres study, we concluded that patients carrying T-275A SNP had significantly lower MPA AUC_0-12_ compared with the noncarriers, and 15% frequency of T-275A obtained in this study was approximately similar to reported frequency of 16.8% in Kuypres study (20). While reported frequency of C-2152T was 12.6% in study reported by Kuypers *et al. *(20), we did not detect this SNP in our population. Lack of detection of some of these mutations has been reported in Japanese and Chinese populations, in whom none of these SNPs were found (13, 22, 23).

In addition to lower MPA AUC_0-12_, due to higher glucuronidation activity of UGT1A9 in carriers of T-275A mutation, C_max_ was significantly lower with no significant difference in C_0_. Since C_0_ is not a good predictor of drug efficacy (24), its influence by polymorphism seems to be of less value than other parameters such as AUC and CL/F. We also failed to show the effect of polymorphism on AUC_6-12 _of MPA as it was reported in Kuypres study (20). The difference between two studies may be related to the type of calcineurin inhibitors received by patients. In Kuypres study patients received tacrolimus which has no effect on enterohepatic recirculation of drug and it only inhibits the glucuronidation of MPA (25-27). On the other hand, our patients received cyclosporine which could interfere with enterohepatic recirculation of drug by the inhibition of MRP-2, and thus diminished the effect of polymorphism on inhibiting enterohepatic recirculation (28-31).

Apparent clearance of drug (CL/F) was significantly higher in T-275A SNP carriers compared to the wild type and there was a positive relationship between the body weight and apparent clearance (r = 0.39, p = 0.01) as reported in other studies (32).

Our patients had higher AUC_ 0-12_ for MPA (104.8±32.3 mg/h/L) compared to other studies. This could be partly because of the effects of other genetic factors such as other concurrent polymorphisms of UGT1A8 and UGT2B7 or SNPs in MRP-2 and non genetic factors such as low steroid dose, ethnicity (33, 34), nutritional habits and concomitant drugs (14). Based on the above results, UGT 1A9 polymorphism can be partly responsible for interindividual differences among the stable renal transplant patients, although most of our patients had acceptable MPA plasma level. Our findings may indicate that routine therapeutic drug monitoring is not necessary in stable renal transplant patients taking MMF. 

## Conclusion

Polymorphism could have a significant impact on the pharmacokinetics of MPA, in particular the AUC0-12 and Cmax. Both parameters were significantly lower while CL/F was significantly higher in the carriers of UGT1A9 polymorphism. Nevertheless, the small sample size of the patients with the UGT1A9 T-275A promoter region single nucleotide polymorphism should be acknowledged in interpretation of the results obtained in this study. More longitudinal studies with a larger number of patients carrying the desired polymorphism are recommended.
